# Social Determinants of Glaucoma Risk and Vision-Related Quality of Life in Rural Nigeria

**DOI:** 10.5334/aogh.5123

**Published:** 2026-05-06

**Authors:** Neil Dogra, Adaora Mezu, BiancaRose Nnabue, Kelechi Mezu-Nnabue, Udo Ubani, Olachi J. Mezu-Ndubuisi

**Affiliations:** 1 Department of Pediatrics, University of Rochester, New York, USA; 2 Mezu International Foundation, Pikesville, Maryland, USA; 3 Abia State University, Uturu, Abia State, Nigeria; 4 Departments of Pediatrics and Ophthalmology, University of Rochester, School of Medicine and Dentistry, 601 Elmwood Avenue, Rochester, New York, NY 14642, USA

**Keywords:** glaucoma, vision-related quality of life, socioeconomic status, global health, social determinants of health

## Abstract

*Background:* Glaucoma is a leading cause of irreversible blindness. Late presentation is common in sub-Saharan Africa; however, most data on social determinants of glaucoma risk originate from high-income countries. We explored how socioeconomic status (SES) and barriers to care affect intraocular pressure (IOP) and vision-related quality of life (VR-QOL) in Imo State, Nigeria.

*Objectives:* To assess relationships between SES, barriers to care, visual functioning, and glaucoma risk in rural Nigeria.

*Methods:* Thirty-six adults at risk for glaucoma were recruited during a free outreach in South-East Nigeria. SES, VR-QOL, and barriers-to-care questionnaires were administered. Participants received medical exams with IOP measurement.

*Findings:* Transportation source was the most consistent SES predictor of outcomes. Participants with more sophisticated transportation sources had lower IOP (*r* = −0.71 [95% CI: −0.92 to −0.17], *P* = 0.019, *n* = 11) and faced fewer barriers to care (*r* = 0.37 [95% CI: 0.04 to 0.63], *P* = 0.026, *n* = 36). Patients with fewer barriers presented with lower IOP (*r* = −0.72 [95% CI: −0.92 to −0.18], *P* = 0.018, *n* = 11) but longer intervals since their last doctor’s visit (*r* = 0.35 [95% CI: 0.01 to 0.62], *P* = 0.036). Longer intervals since the prior doctor’s visit correlated with worse VR-QOL in reading medicine and food labels (*r* = −0.39 [95% CI: −0.65 to −0.07], *P* = 0.017, *n* = 36). Female participants reported greater cost-related, distance-related, and overall barriers to care (*r* = −0.44 [95% CI: −0.67 to −0.11], *P* = 0.008).

*Conclusions:* Transportation limitations and female sex were associated with greater clinical and functional burden. While fewer barriers correlated with lower IOP, longer gaps in care and poorer VR-QOL suggest persistent deficits in glaucoma awareness. Improving transportation access and community health education may support earlier detection and reduce glaucoma burden in rural Nigeria.

## Introduction

Glaucoma is a leading cause of irreversible blindness worldwide [1, 2]. The disease is characterized by gradual damage to the optic nerve from loss of retinal ganglion cells, leading to progressive and irreversible visual field loss [3]. Although the pathogenesis of glaucoma is multi-factorial and not fully elucidated, it is well recognized that glaucoma risk is substantially elevated among individuals with high intraocular pressure (IOP), African ancestry, or a positive family history of the disease [3, 4]. Despite current treatment, glaucoma is often asymptomatic in early stages and remains undiagnosed until significant visual field loss has occurred [3, 5, 6]. This vision loss contributes to substantial functional impairment, diminished quality of life, and long-term economic burden for affected individuals and their families [7–9]. This burden falls disproportionately on low- and middle-income countries, particularly in sub-Saharan Africa, where structural health system barriers delay presentation of glaucoma until irreversible vision loss has set in and quality of life is significantly impaired [10–12]. In Nigeria, 94% of people with glaucoma go either undiagnosed or untreated [10].

In the Owerri region of Imo State, Nigeria, glaucoma was identified as the leading cause of irreversible blindness [11]. Patients with glaucoma also reported substantial reductions in quality of life using the World Health Organization Quality of Life – Brief Version questionnaire, a general instrument that captures physical, psychological, social, and environmental well-being, but not vision-specific functioning [11, 13]. While these findings underscore a clear burden of glaucoma in Owerri, studies have not examined the upstream structural and socioeconomic factors that determine clinical glaucoma risk and its vision-specific functional impacts in this community. This could help channel glaucoma outreach and prevention efforts to the most vulnerable groups.

Mezu International Foundation (MIF), a nonprofit humanitarian organization, conducts annual medical missions in a rural region of Owerri, Imo State, Nigeria. These missions offer free vision screening, chronic disease evaluation, and health education. Over the past decade, MIF has developed and validated community-based surveys to assess key dimensions of health and daily functioning of patients in Owerri—including socioeconomic conditions, chronic disease burden, and vision-related quality of life (VR-QOL) [14–17]. Using questionnaires, this study sought to examine the associations between socioeconomic status (SES), VR-QOL, and barriers to care as they relate to chronic disease among adults at risk for glaucoma in Imo State, Nigeria.

## Methods

This cross-sectional study was conducted on participants of MIF medical mission in Owerri, Imo State, Nigeria. Adult patients between 35 and 85 years old who were at risk for glaucoma were recruited if they met at least one of the following inclusion criteria: (1) self-reported or documented history of glaucoma, (2) IOP > 21 mmHg during on site screening, or (3) a positive family history of glaucoma. Ethics approval was obtained from the Institutional Review Board of Abia State University in Uturu, Nigeria (ABSU/REC/OPT/004/2024). Informed consent was obtained from all participants prior to participation. All aspects of this study were performed in accordance with the Declaration of Helsinki.

### Determination of clinical measures

IOP was measured with a handheld Tono-Pen (Reichert, Buffalo, NY) after administration of a topical anesthetic, proparacaine hydrochloride 0.5% ophthalmic solution (Sigma Pharmaceuticals, North Liberty, IA). IOP was determined in the right and left eyes, of which the higher IOP was used for data analysis.

## Data Collection

Participants completed a written questionnaire with three distinct components. The first component was a modified SES scoring tool adapted from our prior studies, which assessed factors such as housing type, sanitation access, diet quality, and personal asset ownership as shown in Table 1 [14–16]. Next, patients completed a modified VR-QOL questionnaire adapted from our prior studies and based on validated low-vision instruments, focused on functional visual challenges in daily life, reading, mobility, social engagement, and self-care as shown in Table 2 [17]. Lastly, a barriers-to-care questionnaire was developed to evaluate barriers such as cost of visits and medications, distance to the nearest clinic, and ophthalmic health-care literacy as shown in Table 3.

**Table 1:** Modified socioeconomic status questionnaire.

ITEM	RESPONSE OPTIONS	CORRESPONDING SCORE
Education	University Post-secondary/technical Secondary Primary Pre-primary Never enrolled	4 3 2 1 0 −1
Housing type	Own cement (stairs) Own cement (bungalow) thatched/mud Rent Shared home Homeless	4 3 2 1 0 −1
Personal electronics	Radio, TV, cell phone, generator, cable/satellite	+1 for each item owned
Sleeping arrangements	1 per bed 2 per bed 3 per bed Mat/floor >3 per bed	4 3 2 1 0
Drinking water source	Vendor/bottled/borehole Tap/shared borehole Well River/stream Rain	4 3 2 1 0
Toilet facilities	Own (self-flush) Own (bucket) Shared Pit/latrine Public None/bush	4 3 2 1 0 −1
Food access	Restaurant weekly Supermarket weekly None Charity Street aid	4 3 2 1 0
Protein frequency	Daily >3×/week <3×/week Weekly Few/month	4 3 2 1 0
Transportation	Car+AC Car Motorcycle Bicycle Public Walking	4 3 2 1 0 −1
Total SES score	Score range: −4 to 36 Low SES: −4 to 11; medium SES: 12−23; high SES: 24−36	

**Table 2:** Visual-related quality of life questionnaire.

ITEM	YES	NO	DO NOT KNOW
Difficulty reading medicine/food labels	−1	1	0
Difficulty reading newspapers/books/forms	−1	1	0
Difficulty recognizing faces at a distance	−1	1	0
Difficulty visiting friends/family	−1	1	0
Difficulty sewing/cooking	−1	1	0
Difficulty seeing steps/stairs/gutters	−1	1	0
Unable to help with farming/outdoor chores	−1	1	0
Difficulty walking around home at night	−1	1	0
Pain or irritation in eyes	−1	1	0
Need help getting to church/gatherings	−1	1	0
Total VR-QOL score	Sum of all items; range: −10 to 10 **(<−5 low; −5 to 5 middle; >5 high)**		

**Table 3:** Barriers-to-care questionnaire.

ITEM	YES	NO	DO NOT KNOW
Distance to clinic limits seeking care	−1	1	0
Cost of visits limits seeking care	−1	1	0
Cost of medications limits adherence	−1	1	0
First time learning high eye pressure causes vision loss	−1	1	0
Knows difference between optometrist and ophthalmologist	1	−1	0
Total barriers-to-care score	Sum of all items; range: −5 to 5 **(<−2 low; −2 to 2 middle; >2 high)**		

For each component, a total score was generated and graded with higher scores representing better functioning. Thus, for the barriers-to-care instrument, higher scores correspond to fewer reported barriers (i.e., better access), while lower scores indicate greater overall barriers faced in accessing eye care. IOP was collected as part of a broader medical examination.

### Statistical analysis

Descriptive statistics were calculated for age, clinical measures, and questionnaire component scores. Bivariate associations were analyzed using Spearman correlations. All analyses were performed using the GraphPad Prism with significance set at *α* = 0.05. *P* < 0.05 was used to determine statistical significance.

## Results

### Demographics

A total of 36 adults (22 (61%) female and 14 (39%) male) aged 56 ± 12 years participated in this study. Among participants who received IOP examination, mean IOP was 22.36 ± 6.47 mmHg (*n* = 11).

### Socioeconomic status evaluation revealed that unsophisticated transportation correlated with higher risk of glaucoma and poor VR-QOL

**Figure 1:**
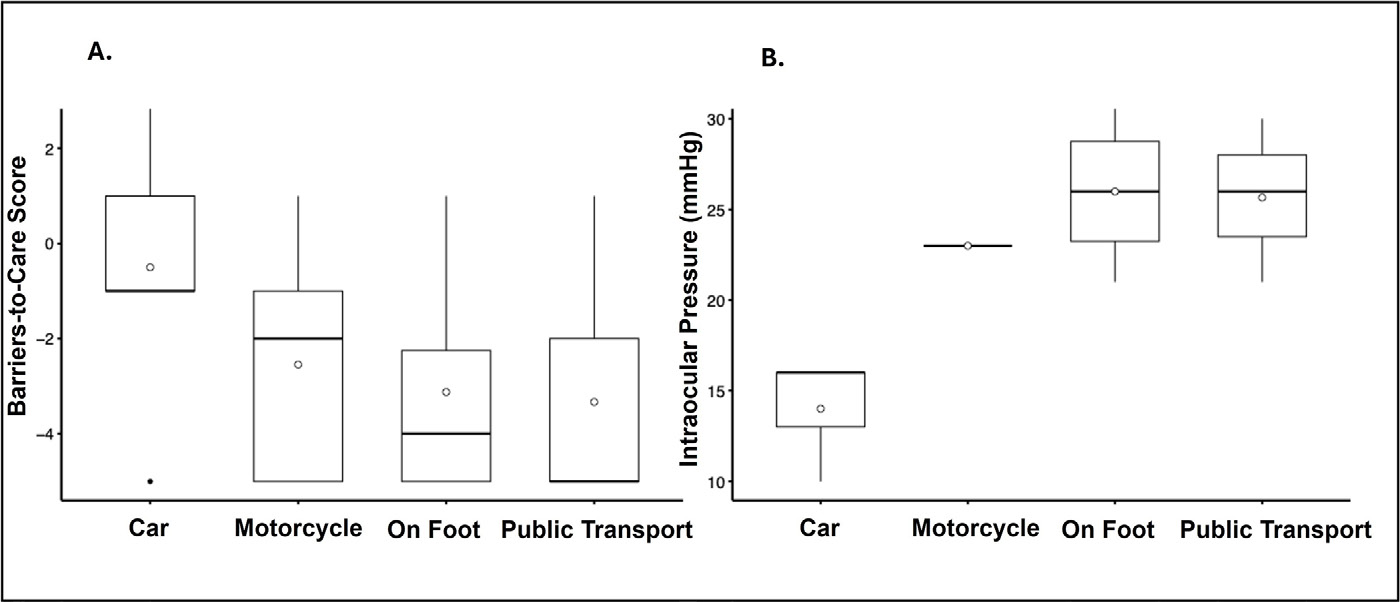
**(A)** Transportation source vs. barrier-to-care scores. Boxplots show barriers-to-care scores across transportation modes arranged by increasing sophistication; medians are represented by black horizontal lines and means by white open circles. **(B)** Transportation source vs. IOP. Boxplots show IOP across transportation modes arranged by increasing sophistication; medians are represented by black horizontal lines and means by white open circles.

SES was not significantly associated with IOP, barriers to care, or VR-QOL. However, transportation access was correlated with various clinical and functional outcomes. Participants using more sophisticated transportation modes, such as cars, had significantly lower IOP (*r* = −0.71 [95% CI: −0.92- to −0.17], *P* = 0.019, *n* = 11) and faced fewer barriers to care (*r* = 0.37 [95% CI: 0.04 to 0.63], *P* = 0.026, *n* = 36). Specifically, participants with cars presented with markedly lower IOP and reported facing fewer barriers to care than those who relied on motorcycles, public transport, or walking (Figure 1). Transportation mode was also associated with greater functioning in several dimensions of VR-QOL. Those with more advanced modes of transportation were significantly less likely to report vision-related difficulty with visiting friends or family (*r* = 0.34 [95% CI: 0.00 to 0.61], *P* = 0.044, *n* = 36), helping with farming or outdoor chores (*r* = 0.35 [95% CI: 0.01 to 0.62], *P* = 0.037, *n* = 35), walking around their home at night (*r* = 0.37 [95% CI: 0.03 to 0.63], *P* = 0.029, *n* = 35), and getting to church or gatherings (*r* = 0.44 [95% CI: 0.11 to 0.68], *P* = 0.008, *n* = 35).

### Sophisticated drinking water sources showed mixed associations with VR-QOL and eye-care utilization

Participants with better water sources were less likely to report difficulty reading (*r* = −0.36, [95% CI: −0.62 to −0.03], *P* = 0.030, *n* = 36). However, participants with more sophisticated water sources such as private borehole over well water or stream were more likely to report that it was their first time learning that high eye pressure can cause vision loss (*r* = 0.38, [95% CI: 0.04 to 0.64], *P* = 0.025, *n* = 35).

### Fewer barriers to care correlated with lower IOP but less frequent health-care utilization

Fewer barriers to care were significantly associated with lower IOP (*r* = −0.72 [95% CI: −0.92 to −0.18], *P* = 0.018, *n* = 11), but poorer VR-QOL (*r* = −0.04 (−0.38 to 0.30), *P* = 0.800, *n* = 36). However, participants with fewer barriers to care also reported longer durations since their last visit to a doctor (*r* = 0.35 [95% CI: 0.01 to 0.62], *P* = 0.036, *n* = 36). Longer intervals since the prior doctor’s visit were associated with functional limitations in VR-QOL, including difficulty reading labels on medicine bottles or food (*r* = −0.39 (-0.65 to −0.07), *P* = 0.017, *n* = 36) and difficulty reading newspapers, books, or forms (*r* = −0.41 (−0.66 to −0.09), *P* = 0.012, *n* = 36).

### Women faced greater cost- and distance-related barriers to eye care

Male participants faced significantly fewer barriers to care (*r* = −0.44 [95% CI: −0.67 to −0.11], *P* = 0.008, *n* = 36) and were less likely to report being limited by distance to clinic (*r* = −0.49 (−0.71 to −0.17), *P* = 0.003, *n* = 35). or medication cost (*r* = −0.46 (’−0.69 to −0.14), *P* = 0.005, *n* = 35) compared to female participants.

## Discussion

This study investigated the associations of SES and barriers to care in adults at risk for glaucoma in rural Owerri, in Imo State, Nigeria. Our study is the first to analyze VR-QOL in association with IOP, a key biomarker of glaucoma risk, in this setting. We provide novel insight into how structural barriers impact the burden of glaucoma risk in this underserved population.

Our findings showed that SES was not significantly associated with IOP, barriers to care, or VR-QOL. This is in contrast with prior studies in Nigeria, where metrics related to SES—such as insurance and education—have been independently linked to eye-care utilization [18, 19]. However, these studies explored the correlations between eye-care utilization to unidimensional metrics of SES, while our scoring tool was an aggregate of several modifiable variables, as shown in Table 1. In addition, Nigeria’s health system remains predominantly cash-based: only 5% of the population is covered by any form of health insurance, and roughly 70% of health spending is paid directly out-of-pocket at the point of service. In such a system, even relatively higher-SES households may face substantial financial barriers, potentially blunting observable associations between SES and glaucoma-related outcomes in our sample [20].

In our study, transportation source, a component of SES, emerged as the most consistent predictor of VR-QOL. Participants with more advanced transportation options reported significantly less barriers to care, lower IOP, and improved VR-QOL, including walking at night, farming, and attending social gatherings. Specifically, access to a personal car—compared with reliance on motorcycles, public transport, or walking—corresponded to markedly lower barriers-to-care scores and substantially lower IOP. This pattern suggests that private car ownership may be a critical threshold enabling eye-care utilization and earlier detection of glaucoma risk. Given that overall SES was not significantly associated with outcomes, this finding suggests that car ownership may represent a more immediate determinant of care access, potentially reflecting mobility or underlying financial stability. These findings align with literature assessing eye-care utilization in rural Nigeria, where transportation availability has been repeatedly identified as a critical factor to timely care [10, 18]. Arinze et al. found that rural Nigerian patients frequently cited transportation costs and lack of accompaniment as key barriers to seeking care, even when symptoms were present [18]. Our study expands on these findings by demonstrating that less sophisticated transportation sources not only predicted greater barriers to care but also higher IOP and impaired VR-QOL. This emphasis on transportation is consistent with findings from higher-resource settings within the United States, where Musa et al. report that transportation limitations substantially hinder glaucoma evaluation and treatment, reinforcing its role as a critical determinant of disease severity [21]. These findings suggest that improving transportation access in rural settings may facilitate access to timely eye care, improving clinical and functional outcomes associated with glaucoma risk.

Water source quality showed mixed associations with visual health. Participants with better water access reported fewer reading difficulties, suggesting that improved living conditions may support visual functioning. However, the greater likelihood of first-time awareness that high eye pressure can cause vision loss also points to persisting gaps in glaucoma education, even among relatively better-resourced households. In this context, advanced water source may reflect greater financial security but not higher health-care literacy. These findings emphasize that environmental advancement alone does not guarantee improved health literacy or outcomes, underscoring the need to coordinate community development initiatives with disease education and preventive screening.

Water source itself may also serve as an upstream factor for glaucoma risk in this community because of its implications on body mineral content. Groundwater serves as a primary drinking-water source for many households in Owerri, and its mineral composition can vary widely, with several groundwater assessments within Owerri reporting concentrations of iron and lead above World Health Organization guideline thresholds, as well as acidic pH levels that can increase the solubility and mobility of metals in groundwater [22].

Such variability raises concern because chronic exposure to water-soluble minerals such as fluoride and to heavy metals including lead have been implicated in glaucoma pathophysiology [23, 24].

Although our study did not measure water quality, our finding that water source correlated with aspects of health-care literacy warrants consideration, as environmental exposures carried through groundwater may intersect with awareness gaps to amplify glaucoma risk. These findings highlight the need to pair infrastructural improvements with strengthened community health education to ensure that upstream environmental factors are recognized and mitigated as part of comprehensive glaucoma prevention efforts.

Participants facing fewer barriers to care had significantly lower IOP, underscoring the role of access in preventing advanced disease presentation. This finding is consistent with an Ethiopian hospital-based case control study from Belete et al., which observed lower IOP among glaucoma patients presenting earlier to care. Obasuyi et al. similarly identified structural factors, such as living distance from hospital and cost of care, were significantly associated with timely entry into the glaucoma care pathway in Nigeria [10]. Our results are also in agreement with findings from Musa et al. in higher-resource settings within the United States, where barriers to care—including financial limitations and low health-care literacy—have been shown to delay glaucoma detection and contribute to worse clinical and functional outcomes [21].

Fewer barriers to care were surprisingly linked to longer intervals since the last clinical visit. These longer gaps were also associated with functional impairments in VR-QOL, such as difficulty reading medicine labels, newspapers, books, or forms. This unexpected result was consistent with findings from Obasuyi et al., who reported that even when eye-care services are geographically available, low glaucoma awareness and inadequate education prevent consistent utilization in rural Nigerian patient populations [10]. It is well documented that health-care literacy, particularly glaucoma awareness, remains markedly limited in rural Nigerian settings; a survey from Osun State found that only 15.8% of adults recognized the term “glaucoma,” underscoring the pervasive knowledge gaps that impede timely care [25]. Evidence from prior MIF missions supports the value of education in improving health behaviors within this rural Nigerian community. Nwaba et al. reported that a brief anemia-education intervention produced significant gains in caregivers’ knowledge and self-efficacy, suggesting that similar community-based approaches may strengthen glaucoma awareness and care-seeking in low-resource settings [14]. The divergence between IOP and VR-QOL further indicates that in settings with limited disease awareness, functional decline may not parallel clinical severity. Because glaucoma is often asymptomatic until late stages, the use of vision-specific functional questions in this study enabled detection of early daily-life limitations that preceded formal diagnosis. Together, these findings emphasize the value of integrating both patient-reported outcomes and clinical measures in community screening programs.

Observed sex disparities in barriers to care, with female participants reporting greater barriers to care and greater cost and distance-related barriers, mirror reports from Olusanya et al. that women in rural Nigeria were significantly less likely to utilize eye-care services due to limited financial autonomy, transportation challenges, and sociocultural restrictions on travel [19]. Obasuyi et al. likewise highlighted gender as a major determinant of unequal access, noting that women face additional structural and financial obstacles that delay glaucoma diagnosis and treatment [10]. Broader studies across Nigeria likewise demonstrate pervasive gender inequities in health-care access, particularly in rural communities where economic dependence and limited transportation compound these barriers [26, 27]. These disparities warrant particular attention, as female sex is an independent risk factor for glaucoma and has been associated with delayed diagnosis and more severe disease at presentation [1].

This study has several limitations, including a modest sample size and limited clinical data, particularly for IOP, which may reduce generalizability and statistical power. The limited number of participants with IOP measurements importantly reflects the practical challenges of obtaining such measurements in broader community-based screening settings, where time, equipment, and personnel constraints can limit comprehensive ophthalmic evaluation. Additionally, knowledge of the distinction between optometrists and ophthalmologists was included as a component of the barriers-to-care measure to assess participants’ ability to identify appropriate sources of eye care. However, in this setting, where provider roles are less clearly differentiated and access is driven by availability and proximity, this distinction may not function as a meaningful barrier to care. The cross-sectional design of this study precludes causal inference. A selection effect is also possible, as the free, open-access nature of the screening may have attracted individuals facing more extreme barriers to care. Nonetheless, these features also represent key strengths. By engaging underserved participants often excluded from clinic-based studies, this design enabled real-world assessment of how structural and socioeconomic factors shape both clinical risk and VR-QOL in a high-need setting.

## Conclusion

Survey findings from a community-based screening conducted during a medical mission in Imo State, Nigeria, show that transportation access and gender were key predictors of both clinical and functional burden associated with glaucoma risk. Participants with better transportation options had lower IOP and higher VR-QOL, while women reported greater cost- and distance-related barriers to care. Notably, some indicators of improved SES or access showed complex associations with worse health-care utilization, indicating that infrastructural improvements alone are insufficient without concurrent efforts to enhance outreach, education, and health literacy. These results highlight the dual importance of physical mobility and equity-focused outreach in mitigating undiagnosed glaucoma and its impact on daily functioning in rural settings. Community health education to improve modifiable health-related behaviors and access to low vision devices to improve visual-related quality of life may be beneficial to rural communities to reduce the burden of glaucoma. Future studies with larger, longitudinal cohorts are needed to confirm causality and evaluate community-level interventions—such as subsidized transport services or targeted outreach programs—that could reduce inequities in glaucoma detection and care.
